# Intermediate-Term Clinical Outcomes After the Shortening Arthrodesis for Ankle Arthropathy with Severe Bone Defect

**DOI:** 10.3390/jcm14134605

**Published:** 2025-06-29

**Authors:** Jae-Hwang Song, Sung-Hoo Kim, Byung-Ki Cho

**Affiliations:** 1 Department of Orthopaedic Surgery, College of Medicine, Konyang University, Daejeon 35365, Republic of Korea; songjajj@hanmail.net; 2 Department of Orthopaedic Surgery, Chungbuk National University Hospital, Cheongju 28644, Republic of Korea; hoo414414@hanmail.net; 3 Department of Orthopaedic Surgery, College of Medicine, Chungbuk National University, Cheongju 28644, Republic of Korea

**Keywords:** ankle, arthropathy, bone, surgery, arthrodesis

## Abstract

**Background/Objectives:** The most common limb-salvage procedure for end-stage ankle arthropathy with severe bone defect is arthrodesis. Successful fusion requires rigid metal fixation, effective filling of the bone defect space, and maximal securing of the contact area between the tibia and talus. In cases with severe bone defect, sufficient grafting using autogenous bone alone is limited, and there is still controversy regarding the effectiveness of allogeneic or xenogeneic bone grafting. This study aimed to evaluate the intermediate-term clinical outcomes after shortening arthrodesis using fibular osteotomy for ankle arthropathy with severe bone defect. **Methods**: Twenty-two patients with shortening ankle arthrodesis were followed up ≥ 3 years. All operations were performed by one senior surgeon and consisted of internal fixation with anterior fusion plate, fibular osteotomy, and autogenous bone grafting. The causes of ankle joint destruction were failed total ankle arthroplasty (7 cases), neglected ankle fracture (6 cases), delayed diagnosis of degenerative arthritis (5 cases), avascular necrosis of talus (2 cases), and diabetic neuroarthropathy (2 cases). Clinical outcomes including daily living and sport activities were evaluated with the Foot and Ankle Outcome Score (FAOS) and the Foot and Ankle Ability Measure (FAAM). Radiological evaluation included fusion rate, time to fusion, leg length discrepancy, and degenerative change in adjacent joints. **Results**: The FAOS and FAAM scores significantly improved from a mean of 21.8 and 23.5 points preoperatively to 82.2 and 83.4 points at final follow-up, respectively (*p* < 0.001). Visual analogue scale for pain during walking significantly improved from a mean of 7.7 points preoperatively to 1.4 points at final follow-up (*p* < 0.001). The average time to complete fusion was 16.2 weeks, and was achieved in all patients. The average difference in leg length compared to the contralateral side was 11.5 mm based on physical examination, and 13.8 mm based on radiological examination. During the average follow-up of 56.2 months, no additional surgery was required due to progression of degenerative arthritis in the adjacent joints, and no cases required the use of height-increasing insoles in daily life. **Conclusions**: Shortening ankle arthrodesis using fibular osteotomy and anterior fusion plate demonstrated satisfactory intermediate-term clinical outcomes and excellent fusion rate. Advantages of this procedure included rigid fixation, preservation of the subtalar joint, effective filling of the bone defect space, and maximal securing of the contact area for fusion. The leg length discrepancy, which was concerned to be a main shortage, resulted in no significant clinical symptoms or discomfort in most patients.

## 1. Introduction

Although ankle arthroplasty is increasingly being selected for patients with severe ankle arthritis, this procedure has well-known relative and absolute contraindications. Severe bone defect in the talus or tibial plafond is considered as one of the poor prognostic factors for total ankle arthroplasty. End-stage ankle arthropathy with severe bone defect can occur in a variety of situations, including progressive bone erosion in osteoarthritis with severe deformities, failed total ankle arthroplasty with periprosthetic osteolysis, neglected ankle fracture, and neuropathic arthropathy [1]. The most common limb-salvage procedure for end-stage ankle arthropathy with insufficient bone stock is arthrodesis [1,2]. In patients scheduled to undergo ankle arthrodesis, severe bone defect refers to a significant loss or deficiency of bone stock that can compromise the ability to achieve a solid, stable fusion between the tibia and talus. Generally, loss of subchondral bone > 50% at the fusion site, and segmental bone loss or cavitary defect ≥ 2cm in diameter are considered the severe bone defect [2]. Severe bone defect can lead to difficulty of achieving bone apposition and compression, delayed union or nonunion, and a requirement for augmentation with a structural graft or custom metal spacer. Successful fusion requires rigid metal fixation, effective filling of the bone defect space, and maximal securing of the contact area between the tibia and talus. In cases with severe bone defect, sufficient grafting using autogenous bone alone is limited, and there is still controversy regarding the effectiveness of allogeneic or xenogeneic bone grafting [3]. Due to insufficient talar bone stock which is not suitable for rigid metal fixation, tibio-talo-calcaneal (TTC) fusion with sacrification of the subtalar joint is usually used. In addition, securing of sufficient contact area between the tibia and talus may be a technically challenging problem, if various ankle deformities are present.

**Figure 1:**
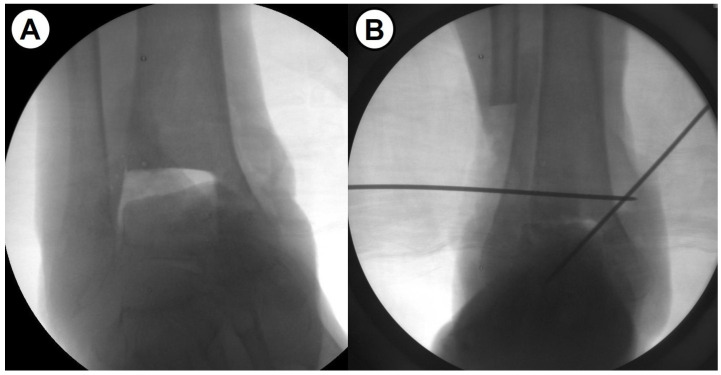
(**A**,**B**) Intraoperative fluoroscopic images show the effects of the fibular osteotomy on ankle fusion.

As one of the solutions for severe bone defect in ankle fusion, fibular osteotomy allows for upward movement of the talus, minimizing the gap and the amount of bone grafting needed, maximizing the contact surface, and optimizing contact area through the correction of coronal plane deformity (Figure 1). We hypothesized that shortening ankle arthrodesis using fibular osteotomy and anterior fusion plate would provide advanced mechanical stability as well as excellent fusion rate. In addition, leg length discrepancy (LLD), which is concerned to be a main drawback of this surgical technique, may demonstrate minimal clinical effects. Given this background, this study aimed to evaluate the intermediate-term clinical outcomes after a novel shortening arthrodesis using fibular osteotomy for ankle arthropathy with severe bone defect.

## 2. Materials and Methods

### 2.1. Study Subjects

**Figure 2:**
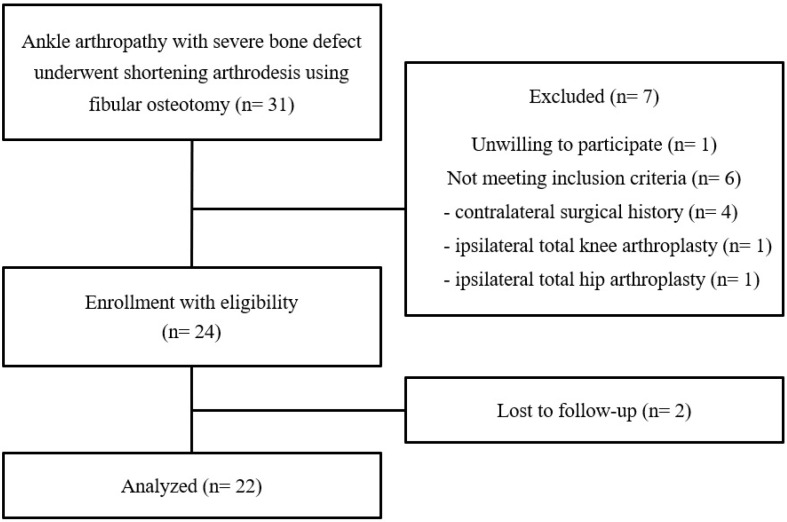
Flowchart diagram of the current study.

We retrospectively reviewed a total of 31 patients treated with shortening arthrodesis using fibular osteotomy for ankle arthropathy with severe bone defect between May 2013 and March 2022. Twenty-two patients were eligible, including a minimum follow-up of 3 years, and analyzed in this study (Figure 2). The causes of ankle joint destruction were failed total ankle arthroplasty (TAA) (7 cases), neglected ankle fracture (6 cases), delayed diagnosis of degenerative arthritis (5 cases), avascular necrosis of talus (2 cases), and diabetic neuroarthropathy (2 cases). The inclusion criteria for this study were as follows: (1) no arthroplasty or arthrodesis history in major joints of contralateral lower extremity, (2) no history of ipsilateral total knee arthroplasty (TKA), (3) no history of ipsilateral total hip arthroplasty (THA), and (4) no concomitant adjacent (subtalar or talonavicular joint) arthritis to need the arthrodesis. The patients with concomitant adjacent joints (subtalar or talonavicular) arthritis needing the arthrodesis were excluded due to the possibility of negative effects on the clinical outcomes. Because of the potential to be a confounding variable on the exact evaluation of leg length discrepancy following the shortening arthrodesis, patients with a history of arthroplasty or arthrodesis major joints (hip, knee, and ankle) of contralateral or ipsilateral lower extremity were excluded. Four patients with contralateral surgical history (2 TKA, 1 TAA, and 1 ankle fusion), one with ipsilateral TKA, and one with ipsilateral THA were excluded. In addition, one patient was unwilling to participate and was excluded from this study. The decision making to perform shortening ankle arthrodesis was based on intraoperative direct inspection for bone defect size. Following the removal of TAA prosthesis or residual cartilage, large bone deficiency more than 2 cm in diameter or height was considered a severe defect, making sufficient filling with cancellous autograft alone difficult.

The study protocol and investigation were conducted with Institutional Review Board approval, and informed consent regarding the use of medical records and radiological data was given to all participants.

### 2.2. Surgical Procedure and Rehabilitation

**Figure 3:**
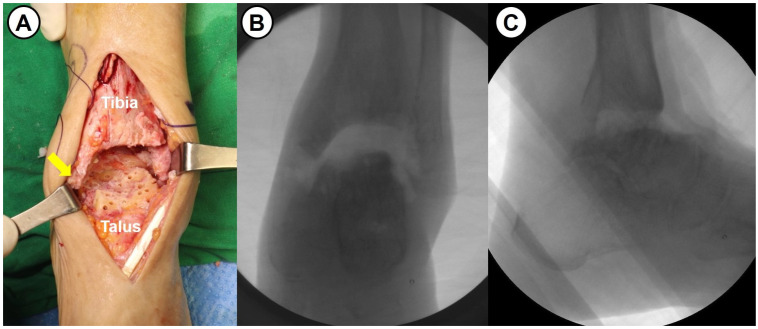
(**A**) Intraoperative photograph shows subchondral preparation (multiple drilling) following the removal of remaining cartilage and necrotic bone in a 63-year-old female patient with avascular necrosis of the talus (arrow: medial malleolus). (**B**,**C**) Intraoperative fluoroscopic images show a large defect space between tibial plafond and talar dome.

**Figure 4:**
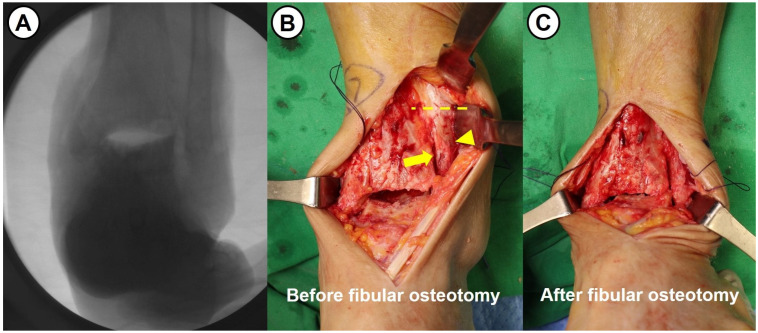
(**A**) Intraoperative fluoroscopic image demonstrating a challenging problem in securing sufficient contact area for ankle fusion. (**B**,**C**) Intraoperative photographs show a difference in defect size between before and after the fibular osteotomy above distal tibiofibular syndesmosis (dotline: fibular osteotomy site, arrow: tibiofibular syndesmosis, arrowhead: fibula).

**Figure 5:**
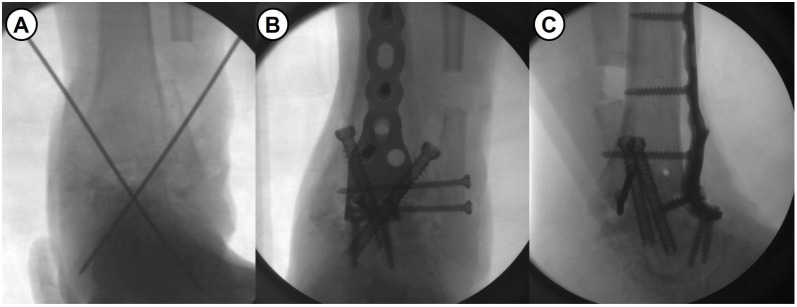
(**A**) Intraoperative fluoroscopic image shows a proximally reposition of the talus and temporary k-wires fixation. (**B**,**C**) With the ankle in neutral position, the rigid fixation with anterior fusion plate and transarticular screws.

All surgeries were performed by a single senior surgeon with over 15 years of experience. Shortening ankle arthrodesis basically consisted of fibular osteotomy, internal fixation with anterior fusion plate, and autogenous bone grafting. A 10 cm sized anterior midline longitudinal skin incision was made and careful soft tissue dissection between the tibialis anterior and extensor hallucis longus tendons was performed to approach into the ankle joint. Following excision of the joint capsule, the remaining cartilage and osteophytes were removed, and sufficient subchondral preparation with multiple drilling to stimulate bone-marrow bleeding was performed (Figure 3). In cases of failed TAA, all prosthesis and metallosis debri were meticulously removed. To maximize the contact area and to minimize the large defect space between tibial plafond and talar dome, fibular osteotomy with protection of the anterolateral neurovascular bundle was performed approximately 1–5 cm above the upper margin of the distal tibiofibular syndesmosis (see Figure 4). Following a transverse osteotomy, the proximal and distal parts were completely displaced, and a segmental bone fragment of about 1–3 cm was harvested from the proximal part if necessary for bone grafting. Then, the distal tibiofibular syndesmosis was dissociated by dissecting the interosseous ligament and anterior and posterior tibiofibular ligaments. The talus was proximally repositioned with maximal compression force. Any remaining defect was filled with an autograft cancellous bone (an average of 11.8 cc) harvested from the proximal tibial metaphysis. With the ankle placed in neutral position, two partially threaded cannulated screws (6.5 mm in diameter) were inserted in a cross-articular manner. Finally, the Titanium Ankle Fusion Plating System (Arthrex, Naples, FL, USA) was secured with locking compression screws (Figure 5). Under C-arm guidance, the final alignment of the ankle and implant position were checked.

A short-leg splint and non-weightbearing ambulation were prescribed for 2 weeks after surgery. Thereafter, cast immobilization following skin suture removal and partial weightbearing ambulation with crutches or walker were maintained up to 6 weeks postoperation. From 6 weeks postoperation, tolerable weightbearing gait with walking boots or removable splint was permitted. Full weightbearing ambulation with normal shoes was allowed when bony union was confirmed clinically and radiologically. A return to sport activities was recommended at least 16 weeks postoperation.

### 2.3. Evaluation of the Patient-Reported Clinical Outcomes

Clinical outcomes were periodically evaluated with the Foot and Ankle Outcome Score (FAOS) [4] and Foot and Ankle Ability Measure (FAAM) [5]. The FAOS comprised of 42 questions and 5 subscales evaluating pain, other symptoms, activities of daily living, sports activities, and quality of life. The FAAM comprised of 2 subscales assessing activities of daily living (21 questions) and sports activities (8 questions). In addition, the visual analogue scale (VAS) for pain during normal walking was evaluated. Zero points indicated no pain and 10 points indicated maximum pain.

### 2.4. Evaluation of Radiological Outcomes

Radiological evaluation included fusion rate, time to fusion, implant-related problem, and degenerative change in adjacent joints. Successful fusion (bony union) was defined as a clinically stable ankle without significant pain (≥3 points on VAS) while walking during daily living, and a radiologically obvious finding of tibiotalar bridging trabeculae and obliteration of the joint space. The definitive conclusion for successful fusion was determined with a computed tomography (CT) scan 3 months postoperatively in all patients. In the current study, bony continuity ≥ 50% of the fusion surface area in three or more slices of CT scan was considered a successful fusion. Patients with unclear bony union underwent a follow-up CT scan every 2 months until complete fusion was determined. Patients without bony union for more than 6 months were diagnosed with delayed union. Patients with nonunion on the CT scan 10 months postoperatively were determined a failed fusion and counselled for revision surgery. The progression of degenerative arthritis in the adjacent joints, including the ipsilateral knee joint and contralateral knee and ankle joints, was evaluated at final follow-up. All radiological assessment were independently performed by two orthopedic surgeons and one radiologist on digital picture archiving and communication system (PACS) imaging system.

### 2.5. Evaluation of Leg Length Discrepancy

**Figure 6:**
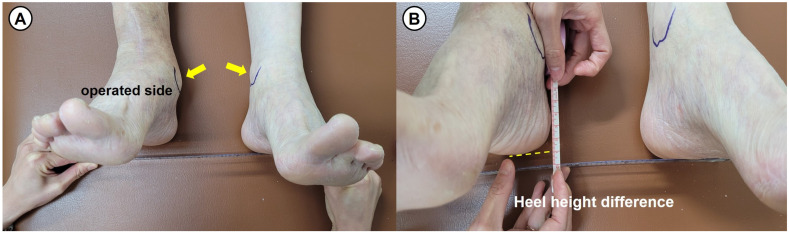
(**A**,**B**) Measurement of the heel height difference to evaluate the leg length discrepancy. Heel height difference indicates a difference in height between both heels of the patient in supine position (arrows: medial malleolus).

**Figure 7:**
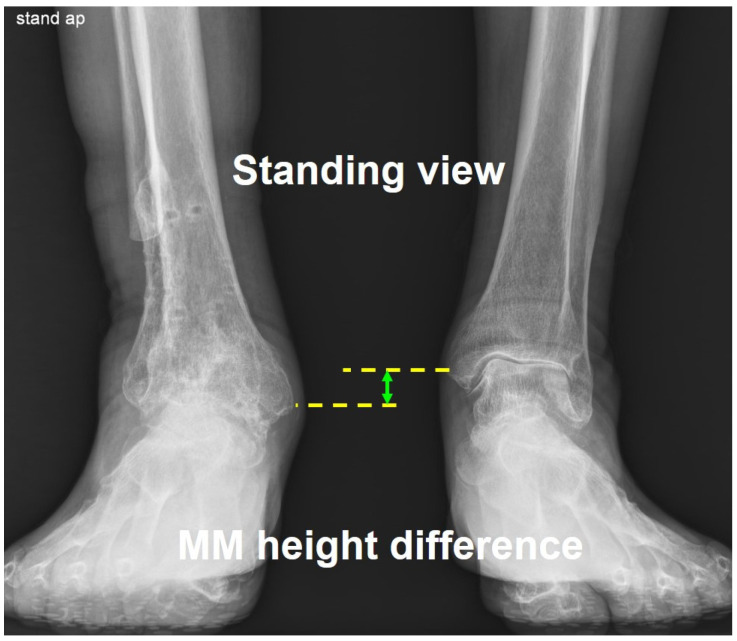
Measurement of the medial malleolar height difference to evaluate the leg length discrepancy. Medial malleolar height difference indicates a difference in height between both medial malleolus in the standing radiograph.

Prior to conducting this study, spine malleolar distance (SMD), long cassette view, and Bell–Thompson scanography were attempted to evaluate the leg length discrepancy. However, we determined that there were limitations in accuracy of these measurement methods. As an indicator of the leg length discrepancy in this study, we used the heel height difference, which measures the difference in height between both heels of patient in supine position (see Figure 6). Additionally, the leg length difference was evaluated radiologically by measuring the height difference between both medial malleolus in the standing radiograph, called the medial malleolar height difference (see Figure 7).

Intraobserver reproducibility of the three researchers was on average 0.86 (range, 0.82–0.91) for the measurement for the heel height difference, and on average 0.92 (range, 0.9–0.95) for the medial malleolar height difference (Table 1). Interobserver agreement between the three researchers was on average 0.84 (range, 0.79–0.89) for the measurement for the heel height difference, and on average 0.87 (range, 0.82–0.91) for the medial malleolar height difference. Therefore, the reliability of the measurement for leg length discrepancy in the current study was found to be excellent.

### 2.6. Statistical Analysis

The statistical analysis was performed using SPSS 22.0 (SPSS Inc., IBM company, Chicago, IL, USA), and *p* value ≤ 0.05 with a confidence interval of 95% was set to statistical level of significance. A normal distribution of all collected data was checked with Shapiro–Wilks and Kolmogorov–Smirnov normality tests. Wilcoxon signed-rank test was used to compare the changes in the patient-reported clinical outcomes between prior to surgery and final follow-up in the same individuals.

## 3. Results

### 3.1. Patient Demographics

The mean age of the patients was 72.6 years (range, 59 to 81 years), and the mean duration of follow-up was 56.2 months (range, 38 to 97 months). This study included 14 male and 8 female patients. The mean volume of the bone defect was 50.4 ± 6.1 cm^3^. The mean body mass index (BMI) was 26.5 kg/m^2^ (range, 23.1 to 28.8 kg/m^2^), and the mean bone-marrow density was 1.05 g/cm^2^ (range, 0.98 to 1.14 g/cm^2^). Bone-marrow density was measured at the hip joint and lumbar spine, and the lower score among the two sites was chosen as the measurement value. Regarding concomitant systemic diseases, there were 6 cases (27.3%) with hypertension, 5 cases (22.7%) with diabetes, and 1 case (4.6%) with rheumatoid arthritis.

### 3.2. Patient-Reported Clinical Outcomes

FAOS significantly improved from a mean of 21.8 points (range, 12–37 points) preoperatively to 82.2 points (range, 69–93 points) at final follow-up (*p* < 0.001). Through comparison in each subscale, there were statistical differences in all 5 subscales between preoperative and final FAOS (Table 2). FAAM score significantly improved from a mean of 23.5 points (range, 13–36 points) preoperatively to 83.4 points (range, 67–92 points) at final follow-up (*p* < 0.001). There were statistical differences in all 2 subscales between preoperative and final FAAM score. VAS for pain during walking significantly improved from a mean of 7.7 points (range, 5–10 points) preoperatively to 1.4 points (range, 0–3 points) at final follow-up (*p* < 0.001).

### 3.3. Postoperative Complications

Regarding early postoperative complications, there were 3 patients (13.6%) of delayed wound healing ≥ 3 weeks, 2 patients (9.1%) of superficial wound infection, and 2 patients (9.1%) of damage to the superficial peroneal nerve (Table 3). All patients with delayed wound healing or superficial wound infection were managed with intravenous antibiotics and continuous dressing care. The tingling sense and numbness on foot dorsum by superficial peroneal nerve injury spontaneously improved within 8 months postoperation. No patient required a blood transfusion due to a significant decrease in hemoglobin level. In total, 2 among 22 patients required intravenous patient-controlled analgesics (PCA) treatment more than twice due to immediate postoperative pain.

Although there was no cases of early implant removal for infection control or wound coverage, in a total of 6 patients (27.3%), hardware removal was performed due to metal irritation symptoms or specific patient needs. During the average follow-up of 56.2 months, no additional surgery was required due to nonunion of the ankle joint or symptomatic progression of degenerative arthritis in the adjacent joints.

### 3.4. Radiological Outcomes

The average time to complete tibiotalar fusion was 16.2 weeks (range, 12–31 weeks), and was achieved in all patients. Only one patient demonstrated delayed union of more than 6 months. Although a progression of degenerative arthritis in the adjacent joints was found in 2 patients (one talonavicular and one subtalar joints), these resulted in no considerable symptoms. There was no patient with symptomatic progression of degenerative arthritis in the contralateral knee or ankle joint. There was one patient with screw breakage and one patient with pull-out of a screw.

### 3.5. The Leg Length Discrepancy

The average difference in leg length compared to the contralateral side at the final follow-up visit was 11.5 mm (range, 5–20 mm) based on measurements of heel height difference and 13.8 mm (range, 8–21 mm) based on radiological measurements of medial malleolus height difference. There was no patient that needed to use height-increasing insoles in daily living.

## 4. Discussion

This retrospective study reports the intermediate-term clinical outcomes after shortening arthrodesis using fibular osteotomy and anterior fusion plate for ankle arthropathy with severe bone defect. The most important finding is that a novel fusion technique has demonstrated satisfactory clinical outcomes and excellent fusion rate, without iatrogenic sequelae secondary to the leg length discrepancy. As one of the solutions to treat end-stage ankle arthropathy with severe bone defect, shortening ankle arthrodesis using fibular osteotomy and anterior fusion plate has a few advantages, including rigid metal fixation, preservation of the subtalar joint, effective filling of the bone defect space (minimizing the amount of bone grafting), and maximal securing of the contact area for fusion. Although definitive evidences based on long-term follow-up are still insufficient, the LLD, which was considered to be an ongoing concern following ankle shortening arthrodesis, brought no significant clinical symptoms or discomfort in most patients. The average LLD compared to the contralateral side was approximately 11.5 mm on physical examination and 13.8 mm on radiological measurement. There was no patient with a need to use height-increasing insoles in daily living.

Nonunion after ankle arthrodesis is one of the most critical problems leading to poor clinical results, and has been reported to happen in approximately 10–15% of patients [6,7]. The risk factors related to nonunion following the ankle arthrodesis include severe segmental bone defect, severe obesity, avascular necrosis of the talus, male gender, smoking, diabetes, severe ankle deformity, major medical comorbidities, prior infection from previous surgery, history of open injury, insufficient surgical technique or fixation, and poor patient compliance [8,9,10,11,12,13,14,15]. With respect to patient-related modifiable risk factors, a recent literature review by Greene et al. indicated cigarette smoking, alcohol overuse, vitamin D deficiency, poor-controlled diabetes mellitus, chronic renal failure, peripheral vascular disease, malnutrition, and immunocompromise [16]. Surgeons should provide sufficient counselling to patients with nonunion risk prior to ankle arthrodesis, and an appropriate perioperative management.

Various metal fixation techniques have been developed to improve the mechanical stability of fusion construct [17,18,19,20,21,22,23,24]. With an increase of popularity in practical use, the biomechanical superiority of anterior fusion plating has been reported [25,26,27,28]. The most critical advantage of anterior fusion plating is that it is known to create a compression force by tension-band effect to fusion site, against a distraction force by ankle plantarflexion [9,22,28]. Tarkin et al. reported that anterior fusion plating combined with transarticular compression screws fixation demonstrated 3.5 and 1.9 times more resistance in the sagittal and coronal planes, compared to the transarticular compression screws alone [28]. In this study, two transarticular compression screws were used to stabilize coronal plane movement (inversion and eversion) and anterior plate fixation was combined to minimize sagittal plane movement (flexion and extension). A systematic review by Kusnezov et al. reported a better fusion rate (97.6%) following supplementary anterior fusion plating [20]. Although there are a few concerns regarding longer skin incision, wound problems due to increased anterior compartment pressure, and soft tissue irritation by the bulky metal implant, anterior fusion plate fixation should be considered for patients with nonunion risk factors [11,20]. Kim et al. have suggested that ankle arthrodesis using an anterior fusion plate in conjunction with tibialis anterior tenotomy may be an effective surgical option for severe ankle arthritis, with excellent fusion rate and lower wound complication rates [29]. Anatomically contoured design of anterior fusion plate allows the locking screws to have more purchase and last longer, even in a talus with large bone defects [9,19,25].

Bone stock deficiency is one of the more challenging problems in ankle arthrodesis. Arthrodesis without a structural graft for large bone defects may result in significant leg length discrepancy and considerable gait alteration. The common causes of a large osseous defect in the ankle joint include avascular necrosis of the talus, failed total ankle arthroplasty, nonunion following arthrodesis, severe comminuted fracture, bony erosion in osteoarthritis with severe deformity, and neuroarthropathy [30,31]. Although autograft bone provides all biologic capacities (osteogenic, osteoconductive, and osteoinductive) necessary for arthrodesis, there are drawbacks, including donor site morbidity, limitation in the amount of harvest, and the need for additional surgical time [32]. Ruland et al. have reported that cellular allograft bone (lineage-committed bone cells within a corticocancellous and demineralized bone carrier) augmentation for ankle arthrodesis may offer an alternative to autograft without donor site morbidity [3]. Clough et al. have reported that a morselized femoral head allograft is a useful option to fill large bone defects, with rapid graft incorporation and no limb shortening [33]. Union rates of structural femoral head allograft have been heterogeneously reported at a broad range, with concerns for graft resorption and infection [34].

Although the clinical and radiological outcomes following various surgical techniques (arthroscopic, open approach, transfibular, TTC fusion, etc.) have been reported by many authors, there is no consensus on the best surgical approach and fixation device. Notably, there are few published studies objectively comparing the results between surgical techniques for end-stage ankle arthropathy with severe bone defect. Although arthroscopic fusion shows the advantages (fast time to union, low complication rates, short hospital stay, and quick postoperative recovery) of a minimally invasive procedure, this technique is known to be ideal for patients with minimal deformity and preserved bone stock. Medial, lateral, or anterior open approaches can provide better visualization for deformity correction and bone graft, despite more soft tissue disruption and hardware-related complications. Despite concerns of higher complication rates and increased risk of nonunion, TTC fusion is used as a salvage procedure to stabilize the entire hindfoot in cases of hindfoot pathology with severe deformity and poor bone quality, failed previous fusion or TAA, and Charcot neuroarthropathy. In terms of fixation devices, Mitchell et al. reported that compression screws alone demonstrated a higher nonunion rate (15.4%) than anterior plate augmentation (7.7%) with compression screws fixation [22]. Another comparative study also reported a better fusion rate (100%) in patients with anterior plate augmentation of compression screw fixation compared to patients (82%) with compression screws alone [35].

Gait rehabilitation after ankle fusion is essential to restore functional walking patterns, reduce compensatory movements, and improve overall mobility [36,37]. In the immediate postoperative period of 6 weeks after surgery, non-weightbearing with a cast or boot is generally maintained and upper body and contralateral limb strengthening exercises are recommended to prevent deconditioning. In the early weightbearing period of 6–12 weeks postoperation, gradual progression to partial and full weightbearing based on radiographic findings is encouraged. In the intermediate rehabilitation period of 3–6 months postoperation, treadmill or overground walking for gait normalization and balance and proprioceptive training on a wobble board are recommended. Moreover, 6 months postoperation, functional training, including stair climbing and walking on an uneven surface, endurance exercises, and return to sport activities, are encouraged. Recently, various mechatronic systems have been developed for gait rehabilitation of patients with an ambulation disability. Ciobanu et al. have shown that these systems allow the physical therapist and medical doctor to manipulate the gait speed, the frequency of steps, and the walking trajectory [38].

This study has some limitations. First, the current study had a retrospective design with a relatively short follow-up duration. A clearer conclusion regarding the progression of degenerative arthritis in the adjacent joints following ankle arthrodesis or implant-related complications may require a longer follow-up evaluation. Second, the current study included a relatively small number of participants, lacking a control group. The question still remains whether metal fixation methods other than an anterior fusion plate result in any difference in the clinical and radiological outcomes. Third, the methods to evaluate leg length discrepancy in the current study were not the measurement techniques used in previously published research. Therefore, there are no validation studies demonstrating the accuracy of these LLD measurements. Measurements of heel height difference on physical examination and medial malleolar height difference on radiographs demonstrated excellent reliability (intraobserver reproducibility and interobserver agreement). However, there may be a debate on the accuracy for LLD measurements, compared to other evaluation methods such as CT scans.

## 5. Conclusions

Shortening ankle arthrodesis using fibular osteotomy and an anterior fusion plate demonstrated satisfactory intermediate-term clinical outcomes and excellent fusion rate. Advantages of this procedure included rigid fixation, preservation of the subtalar joint, effective filling of the bone defect space (minimizing the amount of bone grafting), and maximal securing of the contact area for fusion. The leg length discrepancy, which was considered to be the main limitation, resulted in no significant clinical symptom or discomfort in most patients.

## Figures and Tables

**Table 1:** Analysis of intraobserver and interobserver reliability for leg length discrepancy measurements.

Variables	Intraclass Correlation Coefficient (95% CI *)	
	Heel Height Difference	Medial Malleolar Height Difference
Intraobserver reproducibility		
Researcher 1	0.91	0.95
Researcher 2	0.82	0.92
Researcher 3	0.85	0.9
Interobserver agreement		
Researcher 1–2	0.85	0.88
Researcher 2–3	0.89	0.91
Researcher 3–1	0.79	0.82

* CI, confidence interval.

**Table 2:** Patient-reported clinical outcomes (Wilcoxon signed-rank test).

Scheme 6	Preoperative	PO 6 Months	PO 1 Year	Final F/U	*p*-Value ^†^
FAOS *					
Pain	14.3 ± 8.1	73.9 ± 18.6	83.1 ± 13.2	86.2 ± 11.4	<0.001
Symptoms	31.9 ± 15.3	70.8 ± 17.5	79.5 ± 15.3	83.4 ± 14.2	<0.001
Activity of daily living	30.2 ± 12.9	73.1 ± 15.9	84.2 ± 11.7	89.5 ± 9.6	<0.001
Sports	11.8 ± 6.5	44.5 ± 16.7	62.4 ± 17.5	69.3 ± 16.1	<0.001
Quality of life	20.8 ± 11.2	70.4 ± 16.8	80.2 ± 14.9	82.6 ± 15.2	<0.001
Total FAOS	21.8 ± 9.8	66.5 ± 13.6	77.9 ± 11.4	82.2 ± 13.5	<0.001
FAAM *					
Daily activity	34.5 ± 14.4	71.3 ± 15.4	83.8 ± 11.5	92.5 ± 7.4	<0.001
Sports activity	12.6 ± 6.8	55.9 ± 17.1	68.5 ± 16.6	74.3 ± 15.8	<0.001
Total FAAM score	23.5 ± 10.9	63.6 ± 16.9	76.2 ± 13.4	83.4 ± 12.7	<0.001

Abbreviation: FAOS, Foot and Ankle Outcome Score; FAAM, Foot and Ankle Ability Measure; PO, postoperative; F/U, follow-up. * Data are represented as scores (mean ± standard deviation) changed on the basis of 100 points. ^†^ Comparison between preoperative and final follow-up.

**Table 3:** Complications after shortening ankle arthrodesis using fibular osteotomy and anterior fusion plate.

	Number (%)
Superficial wound infection	2 (9.1%)
Delayed wound healing (≥3 weeks)	3 (13.6%)
Superficial peroneal nerve injury	2 (9.1%)
Metal irritation symptom	2 (9.1%)
Implant removal	6 (27.3%)
Delayed union (≥6 months)	1 (4.5%)
Nonunion (failed fusion)	0
Progressive arthritis in the adjacent joints	2 (9.1%)

## Data Availability

The data presented in this study are available in the article.

## Abbreviations

The following abbreviations are used in this manuscript:

LLD	Leg length discrepancy
FAOS	Foot and Ankle Outcome Score
FAAM	Foot and Ankle Ability Measure
VAS	Visual analogue scale
TAA	Total ankle arthroplasty
